# Genome-scale analysis identifies GJB2 and ERO1LB as prognosis markers in patients with pancreatic cancer

**DOI:** 10.18632/oncotarget.15068

**Published:** 2017-02-03

**Authors:** Tao Zhu, Yuan-Feng Gao, Yi-Xin Chen, Zhi-Bin Wang, Ji-Ye Yin, Xiao-Yuan Mao, Xi Li, Wei Zhang, Hong-Hao Zhou, Zhao-Qian Liu

**Affiliations:** ^1^ Department of Clinical Pharmacology, Xiangya Hospital, Central South University, Changsha 410008, P. R. China; ^2^ Institute of Clinical Pharmacology, Central South University, Hunan Key Laboratory of Pharmacogenetics, Changsha 410078, P. R. China

**Keywords:** pancreatic cancer, GJB2, ERO1LB, prognosis

## Abstract

Pancreatic cancer is a complex and heterogeneous disease with the etiology largely unknown. The deadly nature of pancreatic cancer, with an extremely low 5-year survival rate, renders urgent a better understanding of the molecular events underlying it. The aim of this study is to investigate the gene expression module of pancreatic adenocarcinoma and to identify differentially expressed genes (DEGs) with prognostic potentials. Transcriptome microarray data of five GEO datasets (GSE15471, GSE16515, GSE18670, GSE32676, GSE71989), including 117 primary tumor samples and 73 normal pancreatic tissue samples, were utilized to identify DEGs. The five sets of DEGs had an overlapping subset consisting of 98 genes (90 up-regulated and 8 down-regulated), which were probably common to pancreatic cancer. Gene ontology (GO) analysis of the 98 DEGs showed that cell cycle and cell adhesion were the major enriched processes, and extracellular matrix (ECM)-receptor interaction and p53 signaling pathway were the most enriched pathways according to Kyoto Encyclopedia of Genes and Genomes (KEGG) pathway analysis. Elevated expression of gap junction protein beta 2 (GJB2) and reduced endoplasmic reticulum oxidoreductase 1-like beta (ERO1LB) expression were validated in an independent cohort. Kaplan-Meier survival analysis revealed that GJB2 and ERO1LB levels were significantly associated with the overall survival of pancreatic cancer patients. GJB2 and ERO1LB are implicated in pancreatic cancer progression and can be used to predict patient survival. Therapeutic strategies targeting GJB2 and facilitating ERO1LB expression may deserve evaluation to improve prognosis of pancreatic cancer patients.

## INTRODUCTION

Pancreatic adenocarcinoma is the most common type of pancreatic cancer, accounting for 85% of cases, and is a leading cause of cancer associated death worldwide [1]. The incidence of pancreatic tumors keeps rising although its counterparts of other common tumors are declining [2]. In most cases, symptoms do not show up until the malignancy progresses into advanced stages when surgical resection, the only potential curative therapy, is no longer possible, which leads to a dismal 5-year survival rate [1, 3]. Relapse of the disease after treatment for those 10-20% of patients with resectable tumor also contributes to a poor prognosis [4, 5]. What makes the situation worse is the existence of a subgroup of cancer stem cells (CSCs) within the tumor in that they harbor resistance to chemotherapy and radiation therapy [6–8].

The cause of pancreatic adenocarcinoma is complex and remains to be further elucidated. Smoking and inherited mutations are believed to be two dominant risk factors for this disease [9, 10]. Evolution of pancreatic cancer from precursor lesions to invasive malignancy is marked by accumulating genetic mutations [11]. More than 90% of tumors have oncogenic *KRAS* mutations and more than 50% of cases have inactivation mutations of tumor suppressor genes including *CDKN2A*, *TP53* and *SMAD4* [12–15]. Current knowledge of pancreatic cancer is far from satisfactory to prevent and treat this deadly disease, a better understanding of the underlying molecular events is a prerequisite to improve early diagnosis, efficacy of conventional therapy, and to open new avenues to pancreatic cancer treatment.

Genome-wide profiling offers insights into tumorigenesis and proves to be an efficient way to thoroughly identify pathogenic genes [16]. To investigate the gene expression program and to identify novel targets with therapeutic and prognostic potentials in pancreatic adenocarcinoma at the genome-wide scale, we integrated transcriptome microarray data of five independent pancreatic adenocarcinoma datasets and identified 98 DEGs which were common to all these five expression profiles. After validation of a subgroup of DEGs by using an additional pancreatic adenocarcinoma dataset, we found that *GJB2* were upregulated and *ERO1LB* downregulated in malignant tissues than in normal pancreatic tissues. Patients with higher *GJB2* or declined *ERO1LB* expression had a poorer overall survival rate according to Kaplan-Meier analysis. These results suggested that *GJB2* and *ERO1LB* were potential biomarkers for pancreatic adenocarcinoma whose expression alterations were implicated in development and progression of this malignancy and were associated with prognosis. Therapeutic strategies targeting *GJB2* and facilitating *ERO1LB* expression may deserve evaluation to improve prognosis of pancreatic cancer patients.

## RESULTS

### Identification of DEGs between pancreatic adenocarcinomas and non-malignant tissues

**Figure 1:**
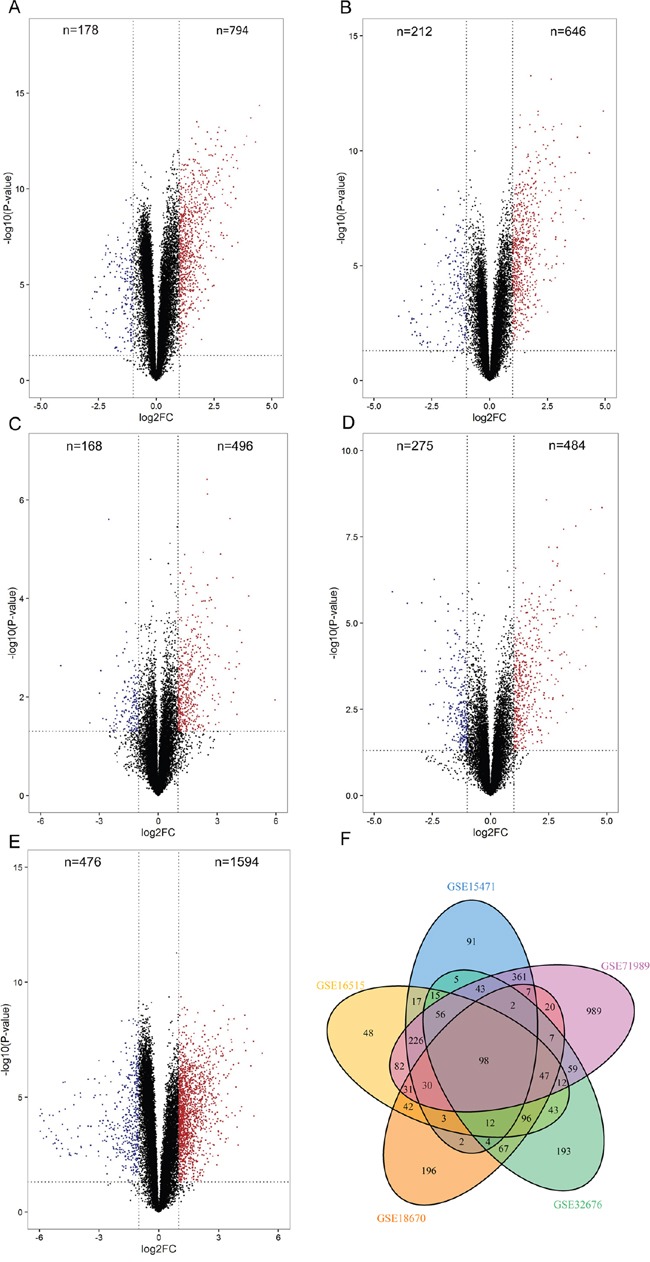
Identification of DEGs between pancreatic adenocarcinomas and non-malignant tissues **A-E**. Volcano plots of differentially expressed genes. 794 genes were identified up-regulated and 178 genes down-regulated in GSE15471 (A), 646 genes up-regulated and 212 genes down-regulated in GSE16515 (B), 496 genes up-regulated and 168 genes down-regulated in GSE18670 (C), 484 genes up-regulated and 275 genes down-regulated in GSE32676 (D) and 1594 genes up-regulated and 476 genes down-regulated in GSE71989 (E). **F**. Venn diagram of the overlapping parts of the five sets of DEGs. Ninety-eight DEGs in total were common to all DEGs sets.

GSE15471, GSE16515, GSE18670, GSE32676 and GSE71989 were used as the discovery datasets for identification of genes differentially expressed in pancreatic cancer. The discovery datasets included 73 normal pancreatic tissue samples and 117 primary tumor samples which were from multiple research sites. Detailed information was listed in Supplementary Table 1. Samples of these five datasets consisted of tumor samples and normal pancreatic samples. Samples in GSE15471 and GSE18670 were pairs including expression data of both tumor and adjacent normal tissues. To investigate gene expression alteration associated with pancreatic cancer progression, we first explored the DEGs of the above five datasets. There were 972 genes (794 genes up-regulated and 178 genes down-regulated) in GSE15471, 858 genes (646 up-regulated and 212 down-regulated) in GSE16515, 664 genes (496 up-regulated and 168 down-regulated) in GSE18670, 759 genes (484 up-regulated and 275 down-regulated) in GSE32676 and 2070 genes (1594 up-regulated and 476 down-regulated) in GSE71989 which were identified as DEGs between normal tissues and tumorous tissues (Figure 1A–1E). Further two-dimensional hierarchical clustering revealed a marked difference of expression modules of the DEGs, with separate clusters between normal and tumor tissues (Supplementary Figure 1A-1E). The intersecting part of the five sets of DEGs consisted of 98 elements. These 98 DEGs were common to all pancreatic tumor samples analyzed and were believed to be relevant in development and progression of this malignancy (Figure 1F), they were listed in Table 1. Among the 98 DEGs, *S100P* (S100 calcium binding protein P) was the top 1 ranked up-regulated gene (Supplementary Figure 2A-2E), in line with previous studies [17, 18].

**Table 1:** Common DEGs identified in pancreatic adenocarcinoma

Regulation	DEGs (Gene Symbol)
Up-regulated	S100P, GJB2, COL5A1, CST1, SLC6A14, IGFBP3, SLPI, SHISA2, JUP///KRT17, DKK1, C19orf33, IFI27, SDR16C5, PPAPDC1A, LAMC2, TMEM158, ITGA2, SFN, S100A6, ANO1, GPRC5A, FOXQ1, SFTA2, AMIGO2, TMPRSS4, ISG15, LY75, PMEPA1, GCNT3, NQO1, TNFRSF21, TSPAN1, KRT19, LAMB3, CD55, PLAC8, MSLN, NMU, EFNB2, S100A2, BIK, LY6E, PMAIP1, MAL2, HK2, SAMD9, OSBPL3, C1orf106, ANLN, KLF5, PI3, MGLL, HN1, CDH3, MLPH, LOC102724257///TMC5, MALL, RACGAP1, MBOAT2, ITGA3, WNT5A, SDC1, FAM83D, AHNAK2, RTP4, MELK, PKM, CDC20, ARNTL2, S100A16, ECM1, CENPK, MTMR11, FAT1, ZWINT, IFI6, SERPINB5, ZG16B, KCNN4, CEACAM5, CEP55, MPZL2, RHPN2, CDC42EP5, PRC1, SPAG1, CCNB1, ABHD17C, AOC1, DLGAP5
Down-regulated	PAIP2B, ERO1LB, IAPP, AF070581, FAM46C, CA4, FAM150B, AOX1

### Functional enrichment analysis of DEGs

**Figure 2:**
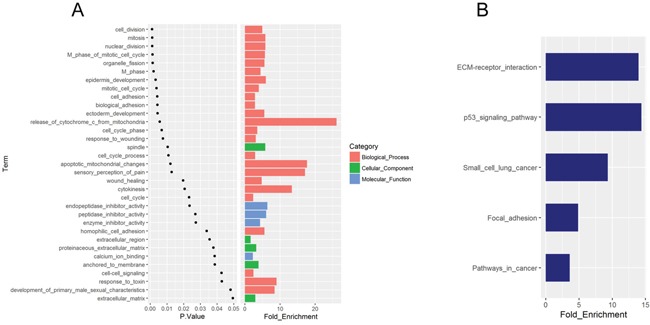
GO and KEGG pathway enrichment analysis of the 98 DEGs **A**. The significantly enriched GO terms. **B**. KEGG pathways significantly enriched, with *P* < 0.05.

To gain insight into the biological roles of DEGs involved in pancreatic adenocarcinoma progression, we performed the GO enrichment analysis and KEGG pathway enrichment analysis. Most of the enriched GO terms belonged to the biological process category. Cell cycle and cell adhesion were the most relevant biological processes, with nearly half of the terms participating in regulating cell cycle and cell adhesion. In terms of cellular component, the enriched GO terms were mainly spindle and extracellular matrix. Of the 4 enriched GO molecular function terms, three were associated with enzyme inhibitor activity (Figure 2A). In addition, KEGG pathway analysis indicated that ECM-receptor interaction and p53 signaling pathway were the most significantly enriched pathways (Figure 2B). It is interesting to note that the ECM receptor interaction pathway mirrored the enriched GO cellular component terms, suggesting that aberrations of extracellular matrix might play an important role in pancreatic cancer progression.

### Validation of DEGs in independent pancreatic adenocarcinomas

**Figure 3:**
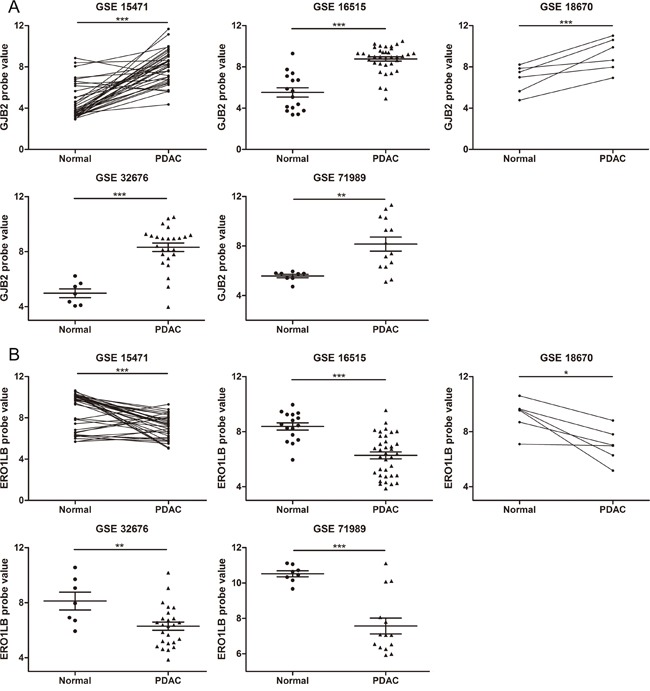
Differential expression of *GJB2* and *ERO1LB* in the discovery datasets **A**. *GJB2* expression was remarkably increased in PDAC than in normal pancreatic tissues. **B**. *ERO1LB* expression significantly declined in PDAC tissues. Samples in GSE15471 and GSE18670 were pairs of adjacent normal and tumor tissue samples. * means *P* < 0.05, ** *P* < 0.01, *** *P* <0.001.

**Figure 4:**
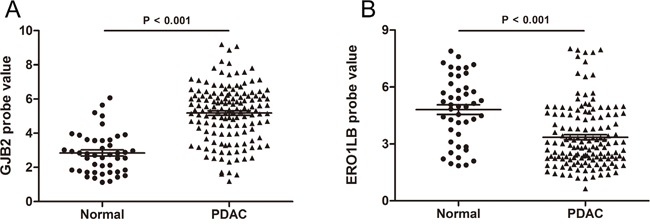
Validation of differential expression of *GJB2* and *ERO1LB* in GSE71729 **A**. Elevated expression of *GJB2* in pancreatic adenocarcinoma. **B**. Reduced expression of *ERO1LB* in pancreatic adenocarcinoma.

To further verify the altered expressions of DEGs in an independent dataset, GSE71729, generated from a different platform, was used as a validation cohort which included 46 normal pancreatic samples and 145 primary pancreatic tumor samples (Supplementary Table 1). Considering that *GJB2* was one of the top 2 ranked up-regulated genes in addition to *S100P* and that *ERO1LB* one of the top ranked down-regulated genes, we focused on these two genes for further analysis in this study. For each of the five discovery datasets, *GJB2* expression was markedly elevated while *ERO1LB* expression significantly reduced in pancreatic adenocarcinoma samples than in control samples (Figure 3A–3B). In consistent with these findings, higher expression levels of *GJB2* and declined levels of *ERO1LB* were seen in the malignant samples of the validation cohort (Figure 4A–4B). These results suggested that *GJB2* and *ERO1LB* disregulation was associated with tumorigenesis of pancreatic adenocarcinoma.

### *GJB2* and *ERO1LB* expression were indicators for prognosis of patients with pancreatic adenocarcinoma

**Figure 5:**
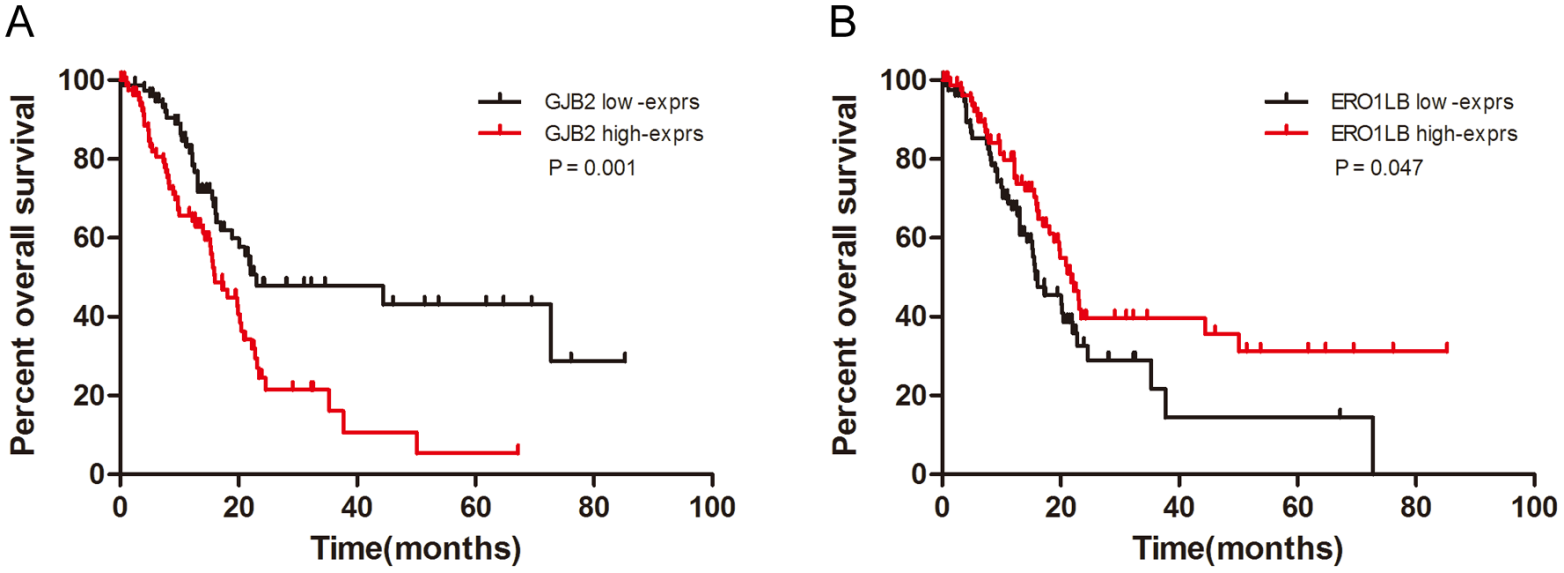
Overall survival curves based on *GJB2* and *ERO1LB* expression **A**. The survival curves of *GJB2* high expression group and *GJB2* low expression group. *GJB2* high expression was associated with poor overall survival (median survival, 16.03 vs. 23.03 months, *P* = 0.001, hazard ratio: 2.082, 95% CI: 1.342-3.230). **B**. The survival curves of *ERO1LB* high expression group and *ERO1LB* low expression group. *ERO1LB* low expression was associated with poor overall survival (median survival, 21.73 vs. 15.77 months, *P* = 0.047, hazard ratio: 0.6417, 95% CI: 0.4141-0.9944). Patients were divided into low- and high-exprs group according to the gene's median probe value.

We further asked whether the elevated expression of *GJB2* or down-regulated *ERO1LB* levels in pancreatic cancer could affect patient survival. Pancreatic adenocarcinoma data with gene expression and clinical information from The Cancer Genome Atlas (TCGA) was used to investigate their prognostic significances. One hundred and sixty-five pancreatic cancer patients were included in this analysis. Their clinical characteristics were summarized in Table 2. Either *GJB2* high expression group or *ERO1LB* low expression group had significantly poorer overall survival (*P* = 0.001, hazard ratio: 2.082, 95% CI: 1.342-3.230, Figure 5A; *P* = 0.047, hazard ratio: 0.6417, 95% CI: 0.4141-0.9944, Figure 5B). The median survival period was 23.03 months for *GJB2* high expression group, and it dropped to 16.03 months in *GJB2* low expression group. *ERO1LB* low expression group had a reduced median survival period, 15.77 months, as compared with the median survival of the high expression group which was 21.73 months. These results indicated that *GJB2* was an adverse factor while *ERO1LB* a beneficial factor for survival of pancreatic adenocarcinoma patients. During the KEGG pathway analysis, we found that integrin subunit alpha 3 (ITGA3), a member of the integrin family which functions as a cell surface adhesion molecule, was involved in 4 of the 5 enriched pathways except the p53 signaling pathway. And its increased expression led to a dismal prognosis (Supplementary Figure 3A-3C), in agreement with a previous report [19].

**Table 2:** Correlation between GJB2, ERO1LB expression and clinicopathologic features of PDAC patients

Factor	Number	GJB2			ERO1LB		
		Low expression	High expression	*P*	Low expression	High expression	*P*
Gender							
Male	91	47 (51.6)	44 (48.4)	0.578	48 (52.7)	43 (47.3)	0.385
Female	74	35 (47.3)	39 (52.7)		34 (45.9)	40 (54.1)	
Age							
≥ 60	114	56 (49.1)	58 (50.9)	0.825	58 (50.9)	56 (49.1)	0.650
< 60	51	26 (51.0)	25 (49.0)		24 (47.1)	27 (52.9)	
TNM stage							
≥ II	144	68 (47.2)	76 (52.8)	0.096	76 (52.8)	68 (47.2)	0.038
I	21	14 (66.7)	7 (33.3)		6 (28.6)	15 (71.4)	
Grade							
III + IV	48	21 (43.8)	27 (56.2)	0.328	23 (47.9)	25 (52.1)	0.770
I + II	117	61 (52.1)	56 (47.9)		59 (50.4)	58 (49.6)	

## DISCUSSION

This study explored modifications of gene expression modules in pancreatic adenocarcinoma at the genome-wide scale by integrating multiple pancreatic cancer transcriptome microarray datasets, which we believe was able to identify alterations at the molecular level with more accuracies, as compared with studies based on a single dataset. For instance, we screened 98 DEGs which were observed in all the pancreatic cancer samples analyzed. Of these DEGs, some were previously recognized regulators associated with this malignancy, such as *S100P* [18], *ITGA3*[19], *ITGA2* [20], *LAMC2* [21], *SERPINB5* [22], *SDC1* [23] and *CST1* [24], some were identified involved in this disease for the first time (Table 1). Further validation of genes with altered expression was based on data from a different platform, which was of benefits in terms of evading platform-specific biases.

Our analysis indicated that pancreatic adenocarcinoma was marked by dysfunctions of cell adhesion, extracellular matrix and cell cycle. The top 3 up-regulated DEGs were all involved in these processes. Cell adhesion dysfunction contributes to, at least in part, the inclination to metastasis of pancreatic cancer, and this propensity is a result of multiple activated signaling pathways in the malignancy [6]. It has been recognized that a dense stroma, with enormous quantities of extracellular matrix in the surrounding area, is a defining characteristic of pancreatic adenocarcinoma [4, 25]. Our gene annotation analysis supported this, considering the enriched cellular components of extracellular matrix and the enriched ECM-receptor interaction pathway.

Up-regulated *GJB2* expression was found at the transcriptional level, in line with a previous immunohistochemical result [26]. In addition, its overexpression was found associated with poorer prognosis. *GJB2* encodes a gap junction protein which plays a role in communication between adjacent cells. In the pancreatic tumor microenvironment, gap junction proteins might facilitate exchange of signals between tumor cells and stroma cells which contributed to progression of the disease, just like their involvement in breast cancer [27]. *GJB2* mutations were frequently identified in hereditary deafness, normal functions of *GJB2* have been believed essential for hearing [28, 29]. ERO1LB is generally endoplasmic reticulum-localized and facilitates generation of oxidative conditions and formation of disulfide bond [30, 31]. It is a pancreas-specific oxidoreductase which was reported to promote insulin biosynthesis and deregulation of which was involved in the pathogenesis of diabetes mellitus [32, 33]. Our analysis revealed that *ERO1LB* was implicated in pancreatic adenocarcinoma whose expression was lower in tumor tissues than in normal pancreatic tissues. Besides, pancreatic adenocarcinoma patients with low *ERO1LB* expression had poorer overall survival than those with high *ERO1LB* expression, suggesting a potential of *ERO1LB* to serve as a prognostic marker. It is interesting to note that up-regulated expression of *ERO1LB* was immunohistochemically detected in pancreatic neuroendocrine tumor (PNET) tissues [34], highlighting different molecular events underlying the two tumor types.

Our study queried the altered gene expression modules of pancreatic adenocarcinoma and identified a set of differentially expressed genes. *GJB2* and *ERO1LB* levels were shown to have prognostic significance and therapeutic strategies targeting *GJB2* and facilitating *ERO1LB* expression may deserve evaluation to improve prognosis of pancreatic cancer patients. However, all our analyses were based on samples from primary tumors, we were not able to clarify the gene expression program of the metastatic tumors. Further efforts are needed to understand the underlying mechanisms of the malignancy, especially mechanisms of the early stages and metastatic tumors.

## MATERIALS AND METHODS

### Pancreatic adenocarcinoma datasets

The discovery and validation datasets were obtained from the Gene Expression Omnibus (GEO,https://www.ncbi.nlm.nih.gov/geo/). The DEGs were identified using five independent pancreatic adenocarcinoma microarray datasets including GSE15471, GSE16515, GSE18670, GSE32676 and GSE71989, with 190 samples in total (117 primary tumor samples and 73 normal control samples). These datasets were generated from the same detecting microarray platform: [HG-U133_Plus_2] Affymetrix Human Genome U133 Plus 2.0 Array. GSE15471 and GSE18670 datasets were composed of matched tumor samples and adjacent non-tumor samples. Data of circulation tumor samples and haematological cells in GSE18670 were not included in this study. GSE71729 dataset including 145 primary pancreatic ductal adenocarcinomas and 46 normal pancreatic samples was used as a validation dataset, the platform of which was Agilent-014850 Whole Human Genome Microarray 4×44K G4112F. TCGA pancreatic adenocarcinoma gene expression data (IlluminaHiSeq) and the associated clinical data was downloaded from the UCSC Cancer Browser (https://genome-cancer.ucsc.edu/) for survival analysis. Data from normal samples and data without follow-up information was removed and finally 165 samples were eligible for survival analysis.

### Data preprocessing

The raw probe-level data (.CEL files) was processed through the robust multiarray average algorithm RMA in the Affy package of R [35]. Expression values were achieved after background correction, quantile normalization and summarizing probe set values into one expression measure. Annotations for the probe arrays were downloaded from GEO. For the cases of multiple probe sets mapping to the same gene, the averages of the probe sets values were taken as the expression values [36].

### DEGs screening and clustering

The limma package was used for identification of DEGs [37]. Genes with |log_2_ fold change (FC)| > 1 and *P* < 0.05 were considered to be differentially expressed between tumors and normal tissues. Hierarchical clustering of the DEGs was based on the Euclidean method which calculates the distance and on complete linkage which is for the tree construction. Visualization of the identified DEGs including volcano plot, venn diagram and heat map was achieved by using ggplot2, VennDiagram and gplots packages of R, respectively.

### Functional enrichment analysis

During functional enrichment analysis of the 98 common DEGs, the online software Database for Annotation, Visualization, and Integrated Discovery (DAVID,https://david.ncifcrf.gov/) was utilized to perform GO analysis and KEGG pathway analysis [38, 39]. Terms with *P* < 0.05 were considered as significantly enriched.

### Statistical analysis

SPSS software (version 22.0, SPSS, Chicago, IL) and GraphPad Prism (version 5, GraphPad Software Inc., San Diego, CA) were used for statistical analysis. Student's *t*-tests were applied for comparisons of two sample groups. Survival analysis was performed through the Kaplan-Meier method and the log-rank test was used to evaluate the statistical significance of the differences. Differences were considered as statistically significant when *P* < 0.05.

## SUPPLEMENTARY MATERIALS FIGURES AND TABLES


