# Supplementary material for: Genome-scale analysis identifies GJB2 and ERO1LB as prognosis markers in patients with pancreatic cancer
Source: Oncotarget. 2017 Feb 3;8(13):21281–9. doi: 10.18632/oncotarget.15068 (PMC5400583; doi:10.18632/oncotarget.15068)
Supplement: Supplementary file 1 [file oncotarget-08-21281-s001.pdf]

Genome-scale analysis identifies GJB2 and ERO1LB as prognosis markers in patients with pancreatic cancer

SUPPLEMENTARY FIGURES AND TABLE

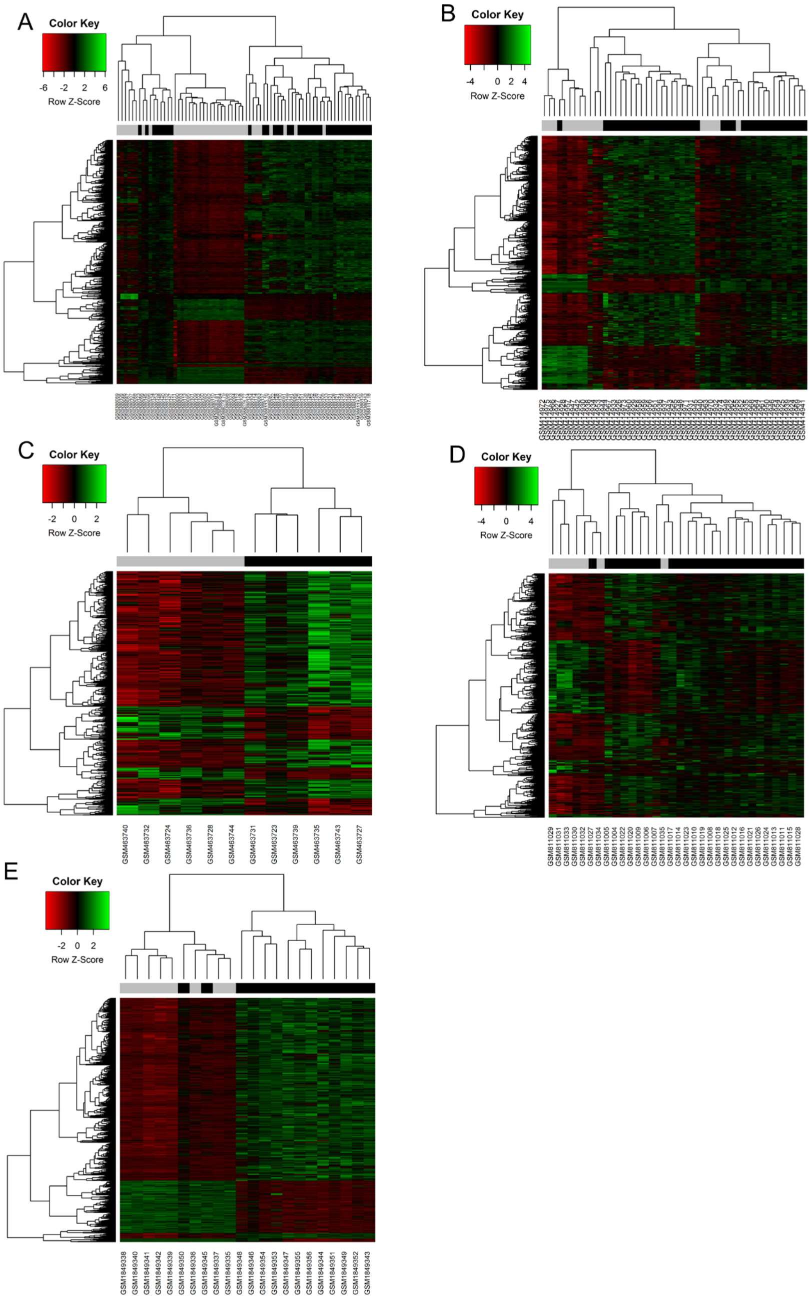

Supplementary Figure 1: Heatmap of gene expression based on two-dimensional hierarchical clustering of the DEGs in the five datasets. **A.** GSE15471; **B.** GSE16515; **C.** GSE18670; **D.** GSE32676; **E.** GSE71989.Genes are in rows and samples are in columns. Normal tissues were represented with gray spots and tumor tissues were represented with black spots.

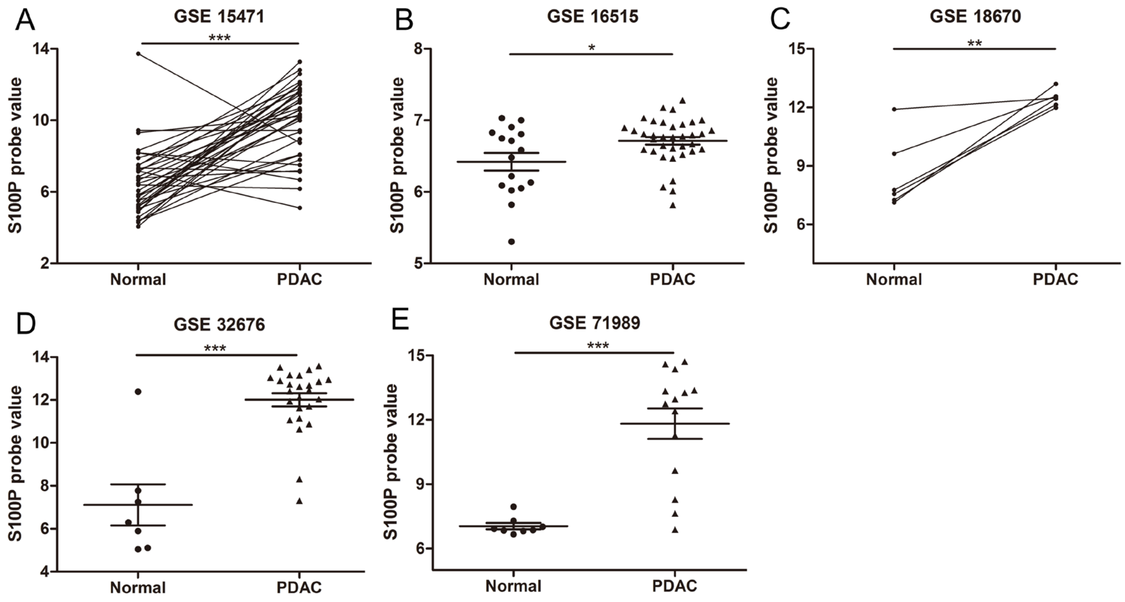

Supplementary Figure 2: Elevated expression of S100P in the five discovery datasets. A. GSE15471; **B.** GSE16515; **C.** GSE18670; **D.** GSE32676; **E.** GSE71989. Samples in GSE15471 and GSE18670 were pairs of adjacent normal and tumor tissue samples. * means $P < 0.05$, ** $P < 0.01$, *** $P < 0.001$.

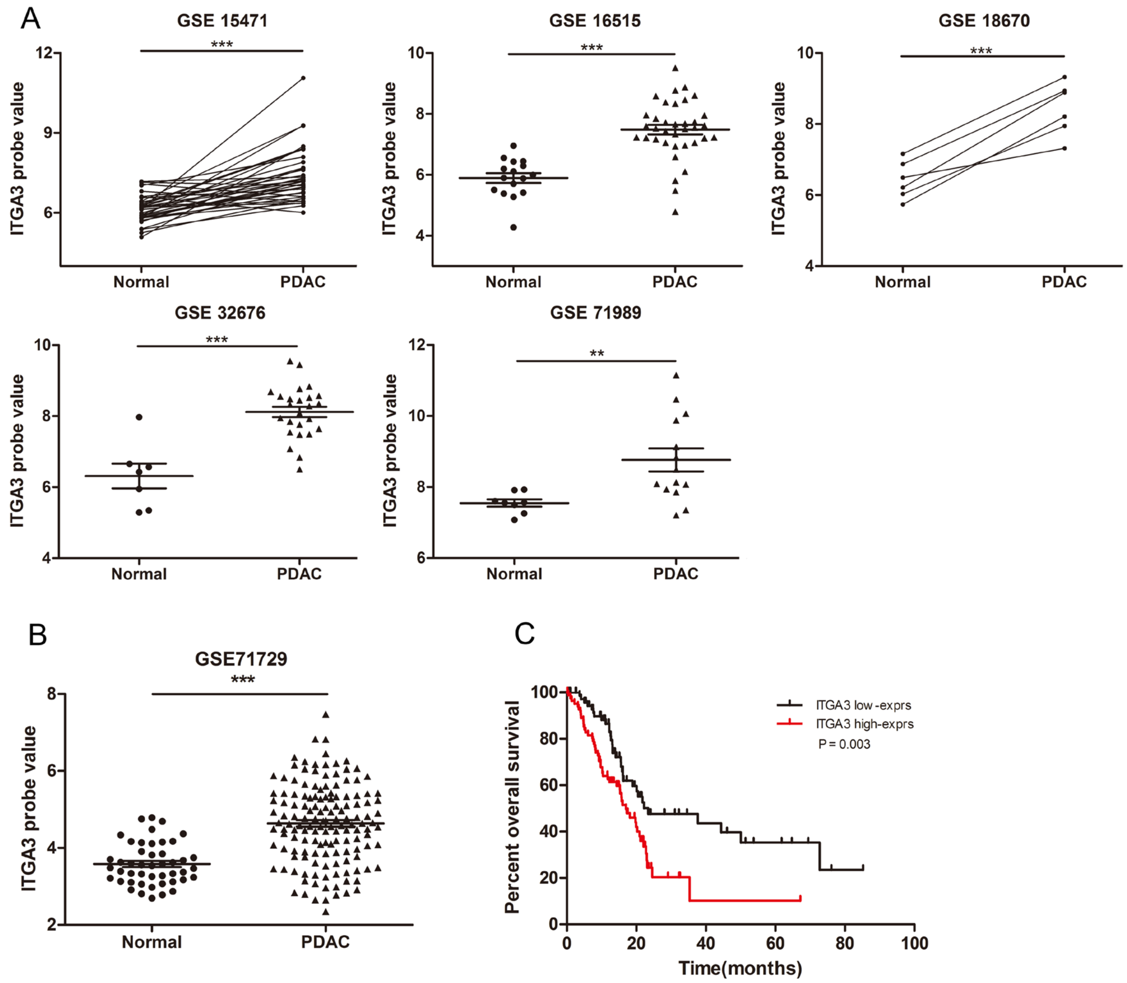

Supplementary Figure 3: Overall survival curves based on ITGA3 expression. A. Elevated expression of *ITGA3* in the discovery datasets. **B.** Increased expression of *ITGA3* in pancreatic adenocarcinoma in the validation cohort. **C.** The survival curves of *ITGA3* low expression group and *ITGA3* high expression group. *ITGA3* high expression was associated with poor survival (median survival, 17.03 vs. 23.40 months, $P = 0.003$, hazard ratio: 1.946, 95% CI: 1.250-3.030). Patients were divided into ITGA3 low- and high-exprs group according to the median probe value of ITGA3.

Supplementary Table 1: List of pancreatic adenocarcinoma datasets used in this study

Dataset	Research Institute	Platform	#Sample		Tumor type	PMID
			#Normal	#Tumor		
**Discovery**						
GSE15471	AI and Bioinformatics, Research, ICI	Affymetrix HG-U133_Plus_2	36	36	PDAC	19260470
GSE16515	Molecular Pharmacology and Experimental Therapeutics, Mayo Clinic	Affymetrix HG-U133_Plus_2	16	36	Pancreatic cancer (not specified)	19732725
GSE18670	Nucleomics Core, Flanders Institute for Biotechnology (VIB)	Affymetrix HG-U133_Plus_2	6	6	PDAC	23157946
GSE32676	Department of Molecular and Medical Pharmacology, University of Los Angeles	Affymetrix HG-U133_Plus_2	7	25	PDAC	22261810
GSE71989	Pharmacy, University of Florida	Affymetrix HG-U133_Plus_2	8	14	PDAC	Not available
**Validation**						
GSE71729	University of North Carolina	Agilent-014850 Whole Human Genome Microarray 4x44K G4112F	46	145	PDAC	26343385

PDAC, Pancreatic ductal adenocarcinoma.
